# First report of aberrant sperm in Edessinae and analysis of the male reproductive system of *Edessa rufomarginata* (De Geer, 1773) (Hemiptera: Pentatomidae)

**DOI:** 10.1371/journal.pone.0311254

**Published:** 2024-12-12

**Authors:** Mauricio da Silva Paulo, Paulo Henrique Rezende, Dayvson Ayala Costa, Ana Clara Pereira Teixeira, Francisco Witallo Sousa do Nascimento, José Lino-Neto, Glenda Dias

**Affiliations:** 1 Departamento de Biologia Geral, Universidade Federal de Viçosa, Campus Universitário, Viçosa, MG, Brasil; 2 Departamento de Entomologia, Universidade Federal de Viçosa, Campus Universitário, Viçosa, MG, Brasil; Alexandria University, EGYPT

## Abstract

Edessinae is one of the ten subfamilies of Pentatomidae, and it is further divided into seven genera. Among these, *Edessa* Fabricius, 1803 is the most diverse genus, boasting around 300 species recognized for their ecological and economic significance worldwide. The inclusion of various pentatomids in the *Edessa* genus has led to mistakes in its taxonomy due to common morphological features and misidentifications. An alternative to avoid mistakes is to use diverse datasets to characterize and classify insects, such as the male reproductive system and sperm morphology, for their variability and conserved traits within a clade. Thus, we described the morphology of the male reproductive system, spermatozoa, and spermiogenesis of *Edessa rufomarginata* (De Geer, 1773) using light microscopy. We discovered that their male reproductive system consists of a pair of elongated testes with four follicles each. The analysis revealed for the first time the presence of dimorphic spermatozoa in Edessinae. There are two distinct morphotypes: spermatozoa type I, produced by follicles 1, 2, and 3, with a total length of approximately 325 μm and a nucleus of 34 μm and spermatozoa type II, produced by follicle 4, measuring approximately 156 μm in total length and 73 μm in the nucleus, and showing an aberrant sperm morphology with different morphology from what has been described in Pentatomidae. The presence of sperm dimorphism in *E*. *rufomarginata* are not reported in any other Pentatomidae to date, and it may contribute to establishing taxonomic limits within the subfamily Edessinae.

## Introduction

Edessinae is one of the ten subfamilies of Pentatomidae [1, 2] and is subdivided into seven genera, including the richest genus, *Edessa* Fabricius, 1803 [3, 4], with around 300 species described [5]. However, due to the lack of clear generic boundaries, it has been referred to as a “repository of species” [6] or a “garbage group” [5], where species of Edessinae with no clear taxonomy position are included in this genus. According to Fernandes and Doesburg [7], many pentatomids have been erroneously identified as belonging to *Edessa* due to their generalist characteristics and identification errors, incomplete descriptions of specimens, variations in species in different regions, and difficulties in obtaining the holotypes are the factors contributing to this “state of chaos” in their taxonomy.

Since Pendergrast’s [8] work on the heteropteran reproductive apparatus and classification, this type of data, especially on the male reproductive system, has been used to characterize and classify certain groups based on their specific shared traits. The reproductive system in Heteroptera consists of a pair of testes that are covered by a pigmented connective capsule, a pair of vasa deferentia that contain seminal vesicles, an ejaculatory bulb, a common ejaculatory duct, and paired accessory glands. The number of testicular follicles in each testis can range from one to nine [8–16].

Insect sperm have been used in several studies to characterize groups. Their morphology and ultrastructure can provide valuable insights into evolutionary relationships, reproductive biology, and taxonomic classification within insect species [17–19]. Spermatozoa show notable morphological variation, even at the species level, related to size, shape, number of components, and ultrastructural organization [17]. Thus, it can and has been used in the systematics of different groups of insects, including Pentatomidae [11, 20].

Based on morphological variations observed in Pentatomidae sperm, Bowen [20, 21] found that this group of stink bugs produces polymorphic sperm (two or more sperm morphotypes developed in the same male individual). Sperm polymorphism in Pentatomidae can be explained by the “harlequin” lobe theory [22, 23]. Schrader [22, 23] proposed that meiosis occurs differently in one of the testicular follicles, resulting in aberrant spermatids (with a different number of chromosomes) [22–24]. These variations lie in the presence of more than one sperm class varying in size and cellular ultrastructure [17].

In this study, we described the morphology of the male reproductive system and the morphology of the spermatozoa of *Edessa rufomarginata* (De Geer, 1773). The analysis of these data in *Edessa* can provide additional evidence for understanding their reproductive biology, evolution, and taxonomic classification since sperm in Pentatomidae have unique characteristics.

## Materials and methods

Adult individuals, males, and females of *E*. *rufomarginata* were collected between April and September 2022 in cultivated *Solanum* spp. in the municipalities of Viçosa (-20.759684, -42.862992) and Diamantina (-18.223516, -43.612088), Minas Gerais state, Brazil.

Ten males and eight females were dissected in 0.1 M sodium phosphate buffer pH 7.2 (PBS). To examine the histology of the testes and accessory glands, they were fixed in 2.5% glutaraldehyde + sucrose 3% in PBS for 24 hours and Carnoy solution (Ethanol 95% + Glacial acetic acid = 3:1), respectively. Then, the samples were washed in the same buffer, post-fixed in 1% osmium tetroxide (the accessory glands did not go through this stage), dehydrated in an increasing series of ethanol (30%, 50%, 70%, 90%, and 100%) and included in Historesin® plastic resin (Leica Historesin, Heidelberg, Germany) and polymerized at 60°C for 12 hours. Semi-thin sections (1.0–1.5 μm for the testicles and 2.0–2.5 μm for the accessory glands) were obtained with glass knives on a Leica RM 2255 automatic microtome and mounted on histological slides. The sections of the testes were stained with a 1:5 filtered Giemsa solution in distilled water for 30 minutes. Sections of the accessory glands were subjected to acid hydrolysis with Periodic Acid for 25 minutes, washed in distilled water, and stained with Shiff’s Reactive (P.A.S.) for 1 hour.

For sperm analysis, the vasa deferentia from males and the spermatheca from females were transferred to histological slides with a drop of PBS, where it was smeared. After drying at room temperature, the samples were washed with distilled water, dried again at room temperature, and stained with Giemsa. Some samples were stained with DAPI solution (0.2 mg/mL) + mounting medium and covered with a coverslip to determine the size of the nuclei. All samples (sperm slides and histological sections) were photographed using a photomicroscope (Olympus, BX-60, Olympus Corporation, Tokyo, Japan) with an Olympus Q-Color3 digital camera attached. After completing these steps, we used thirty sperm of both morphotypes from each male for measurements using the software Sperm-Sizer-1.6.6 [25]. All materials used in the research remain stored at room temperature in the Cellular Ultrastructure Laboratory, at the Federal University of Viçosa, Minas Gerais, Brazil.

## Results

### Anatomy of the male reproductive system and spermatozoa

**Fig 1:**
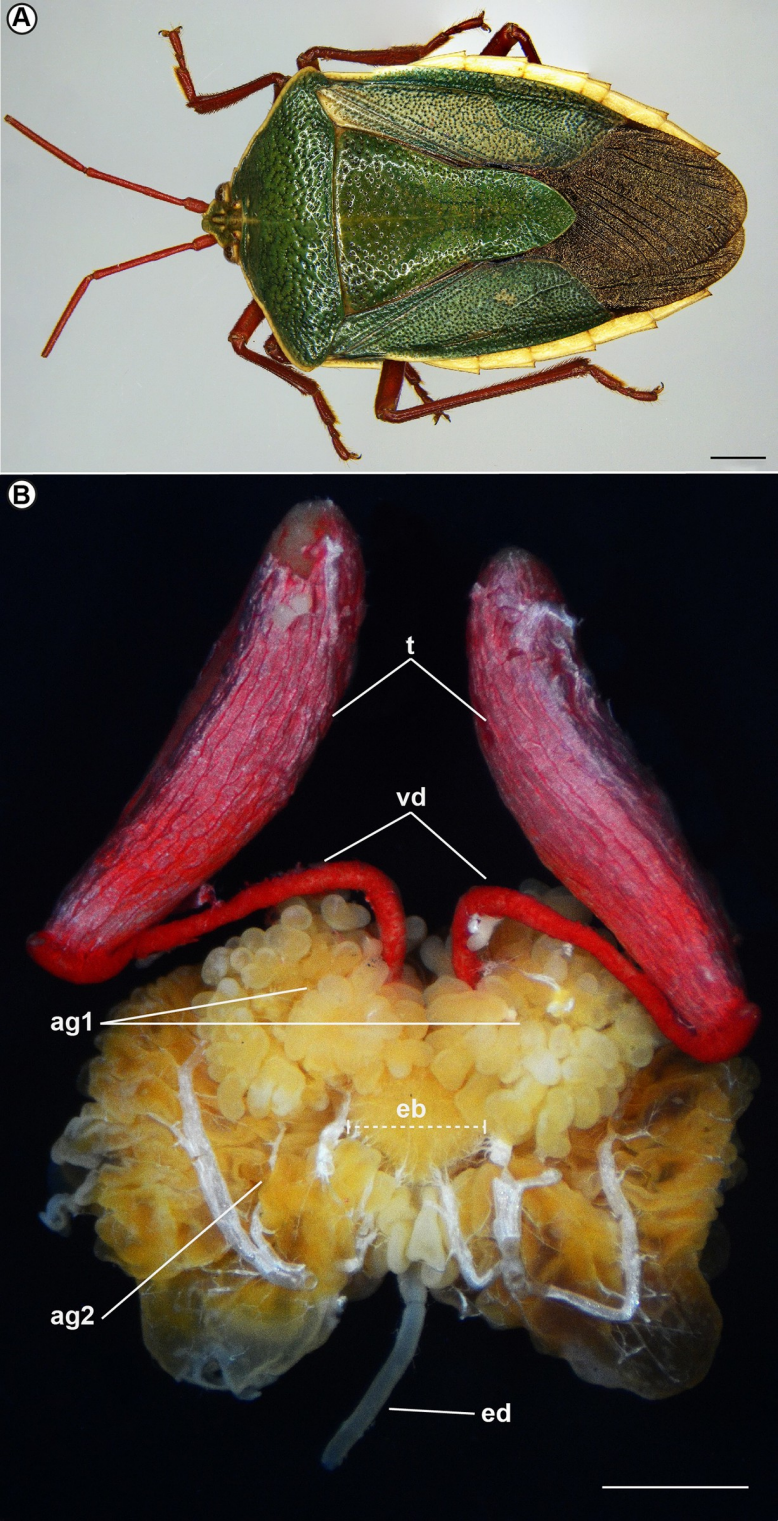
Male individual of *Edessa rufomarginata* (A) and its reproductive system (B). The male reproductive system of *E*. *rufomarginata* is composed of a pair of elongated testes (t), followed by a pair of long vas deferens (vd). Note the red-pigmented peritoneal sheath covering both structures. Two types of accessory glands (ag1 and ag2) are also observed, in addition to the ejaculatory bulb (eb) in the central region between the accessory glands. In the distal portion is the ejaculatory duct (ed). Scale bars: A: 2 mm; B: 1,5 mm.

Males of *E*. *rufomarginata* (Fig 1A) have a pair of elongated testes subdivided into four follicles. Adjacent to the testes are the vasa deferentia, which converge into an ejaculatory bulb and, subsequently, into an ejaculatory duct. Following the ejaculatory duct is the aedeagus. It is interesting to note that *E*. *rufomarginata* does not have dilated seminal vesicles. In this species, the vasa deferentia are relatively thick (nearly 0.5 mm in diameter and around 4.5 mm in length) and serve as sperm reservoirs until copulation. Two types of accessory glands are also present. One type consists of small paired accessory glands, while the second is a singular, more prominent, and intrinsically coiled gland, presenting long ducts with secretory activity. The testes and vasa deferentia are surrounded by red-pigmented connective tissue capsules (Fig 1B).

**Fig 2:**
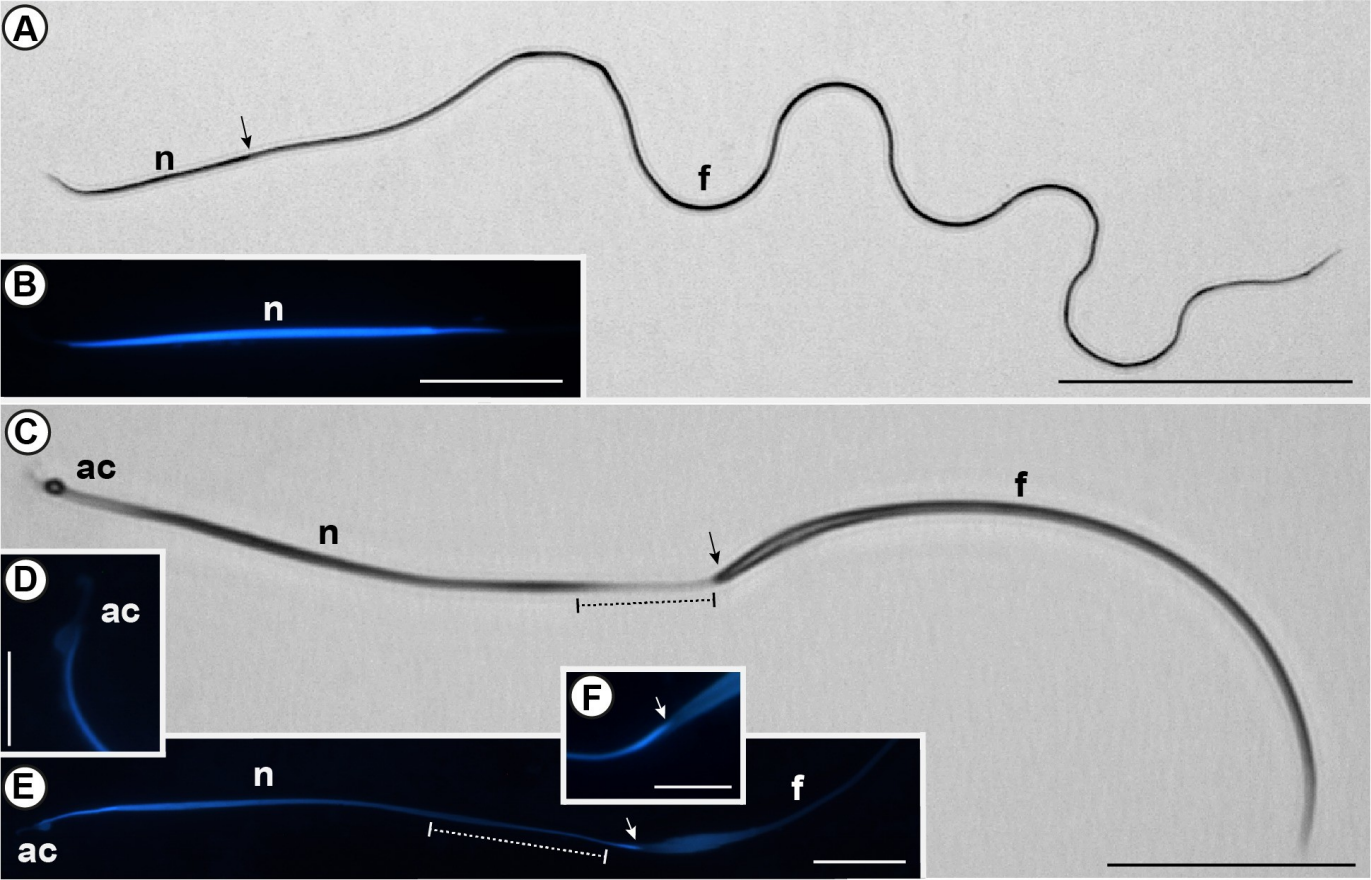
Spermatozoa of *Edessa rufomarginata*. (A): Spermatozoa type I evidenced by Giemsa stain and (B): nucleus (n) stained with DAPI. (C): Spermatozoa type II evidenced by Giemsa stain. (D): detail of acrosome (ac) and (E): nucleus (n) stain with DAPI. Note the nuclear region with low chromatin condensation (dashed lines) in C and E. (F): nuclei-flagellum transition (arrow) showing the final region of the nucleus with highly condensed chromatin inserted into the flagellum (f). Scale bars: A: 50 μm; B and E: 10 μm; C: 25 μm; D and F: 5 μm.

Spermatozoa extracted from the vas deferens exhibited dimorphism, meaning there are two distinct cell morphotypes easily distinguishable under light microscopy (Fig 2A–2D). In each testis, testicular follicles 1, 2, and 3 produce spermatozoa type I. These thin and elongated cells were the only ones found in the female’s spermatheca. They are approximately 325 μm long, with a nucleus length of 34 μm (Fig 2A and 2B). On the other hand, spermatozoa type II originates exclusively from testicular follicle 4. These spermatozoa are shorter, measuring approximately 156 μm in length and 73 μm in nucleus length (Fig 2C and 2D). These sperm seem flattened, with notable dilations in both the nucleus and the flagellum. The acrosome was on the anterior tip of the nucleus, showing lighter staining in the techniques used and exhibiting a globular region positioned lateral to the tip of the nucleus (Fig 2C–2E). Notably, the nucleus of this sperm demonstrates variations in its structure, with some nuclear regions exhibiting more condensed chromatin, resulting in more pronounced dilation compared to areas where the chromatin is less condensed (Fig 2E). Furthermore, the end of the nucleus shows high chromatin condensation, and it appears to be a dilation in the nucleus-flagellum transition zone (Fig 2E and 2F).

### Histology of the male reproductive system and spermatogenesis

**Fig 3:**
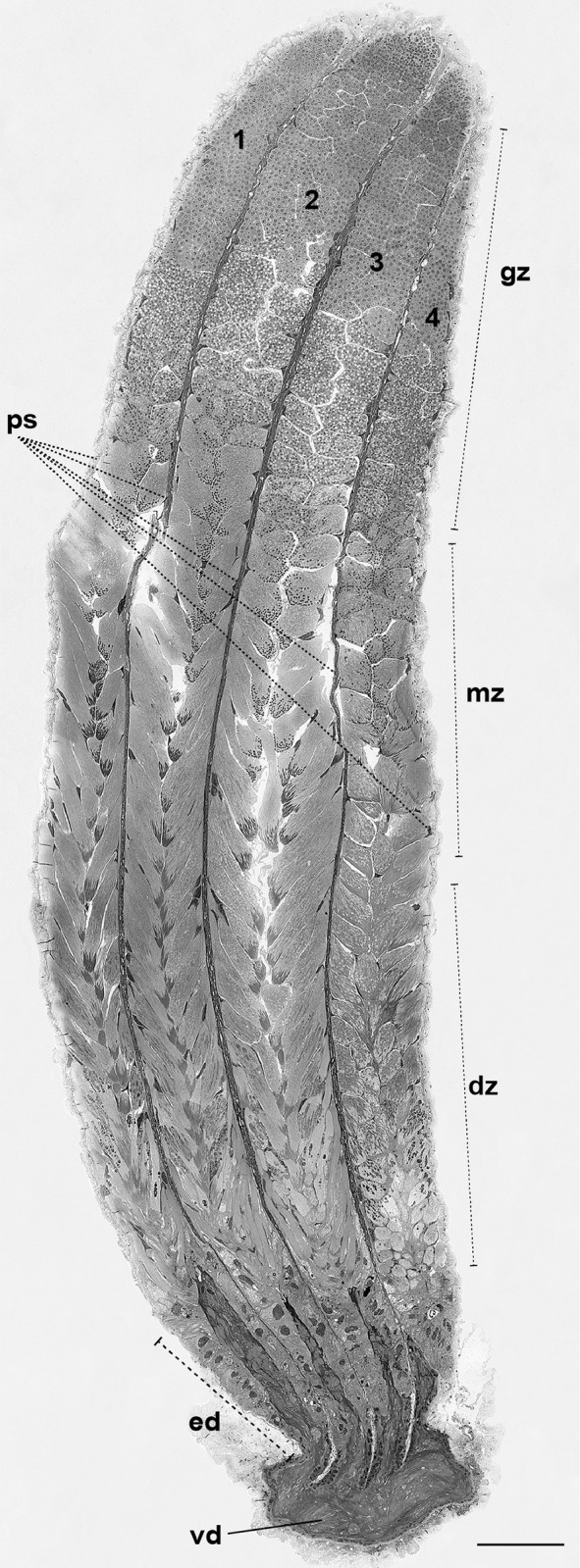
Longitudinal section of one of the testes of *Edessa rufomarginata*. The testes comprise 4 testicular follicles (1, 2, 3, and 4) and are covered externally by the peritoneal sheath (ps). This sheath also internally separates the 4 follicles. Spermatozoa are organized into cysts. In the apical region of the follicles are the initial cysts, in the growth zone (gz); in the medial portion, the sperm cells advance to the maturation zone (mz); and in the distal region, the final cysts are in the differentiation zone (dz). At the end of their development, the spermatozoa are released into the efferent ducts (ed). The latter extends to the vasa deferentia (vd). Scale bar: 175 μm.

Longitudinal sections of testicular follicles of *E*. *rufomarginata* provide information about the process of spermatogenesis in male adult individuals. Cysts in various stages of germ cell differentiation were observed (Fig 3), indicating an ongoing process of spermatogenesis into adulthood. Despite the similar elongated morphology of all four testicular follicles, follicle 4 appears shorter (Fig 3). However, even with these length differences, the spermatogonia undergoing initial differentiation are consistently located in the apical region of all follicles. In contrast, mature sperm are found in the basal part of the testes, situated close to the efferent ducts (Fig 3). This organization suggests spatial differentiation along the longitudinal axis of testicular follicles, where germ cells progress through various stages of development, from the apical to the basal region. The continuous process of spermatogenesis in adult *E*. *rufomarginata* could indicate constant sperm production.

**Fig 4:**
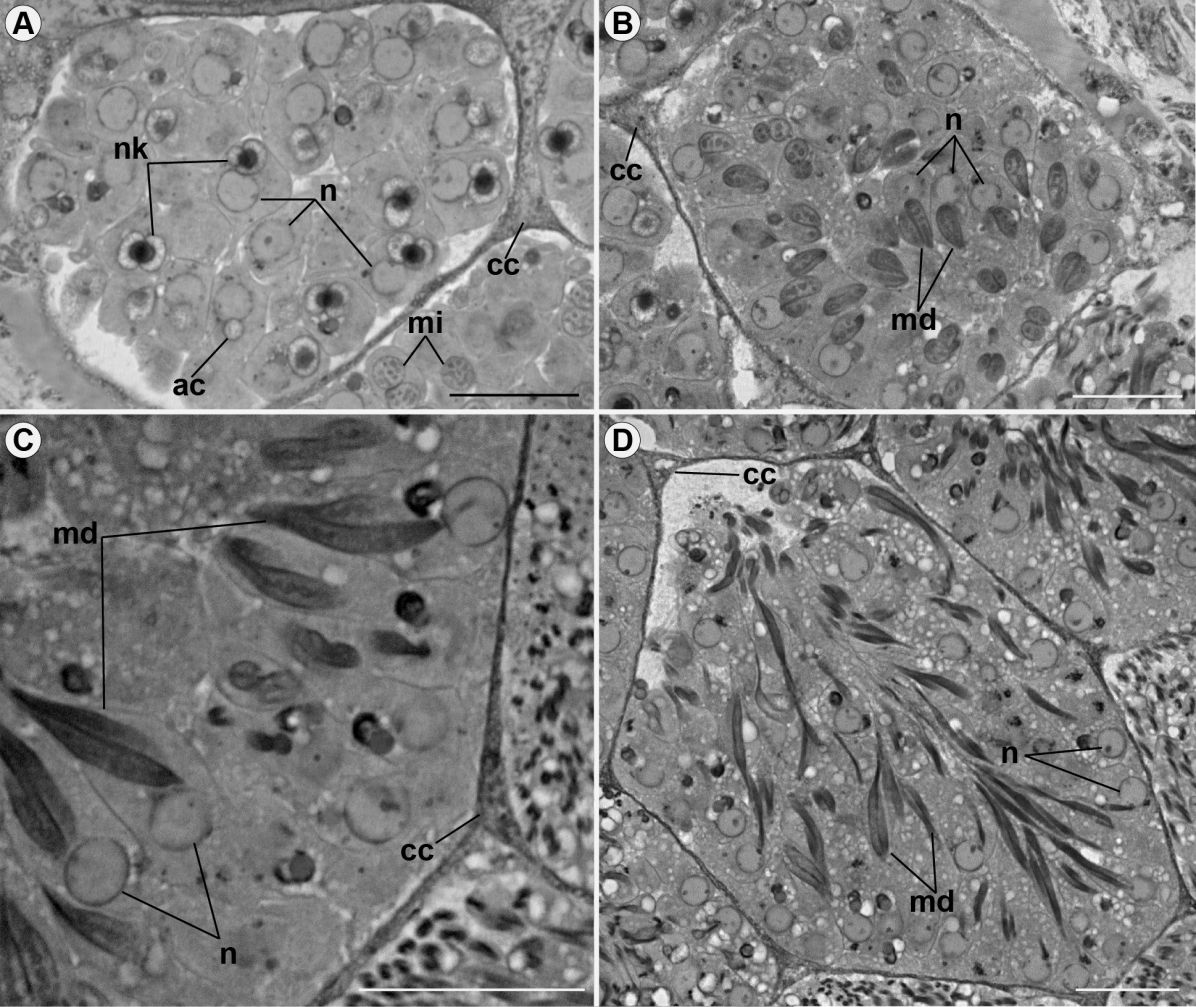
Details of spermiogenesis of spermatozoa type I in *Edessa rufomarginata*. (A): cysts with developing spermatids, a stage marked by the fusion of mitochondria (mi), forming nebenkerns (nk). Note the rounded nuclei (n) in the four images. In (B), (C), and (D), the nebenkerns (nk) fission takes place, originating the mitochondrial derivatives (md), now in pairs. Mitochondrial derivatives (md) become elongated as the flagellum elongates. cc: cyst cell nuclei; ac: pre-acrosomal vesicle. Scale bars: 20 μm.

**Fig 5:**
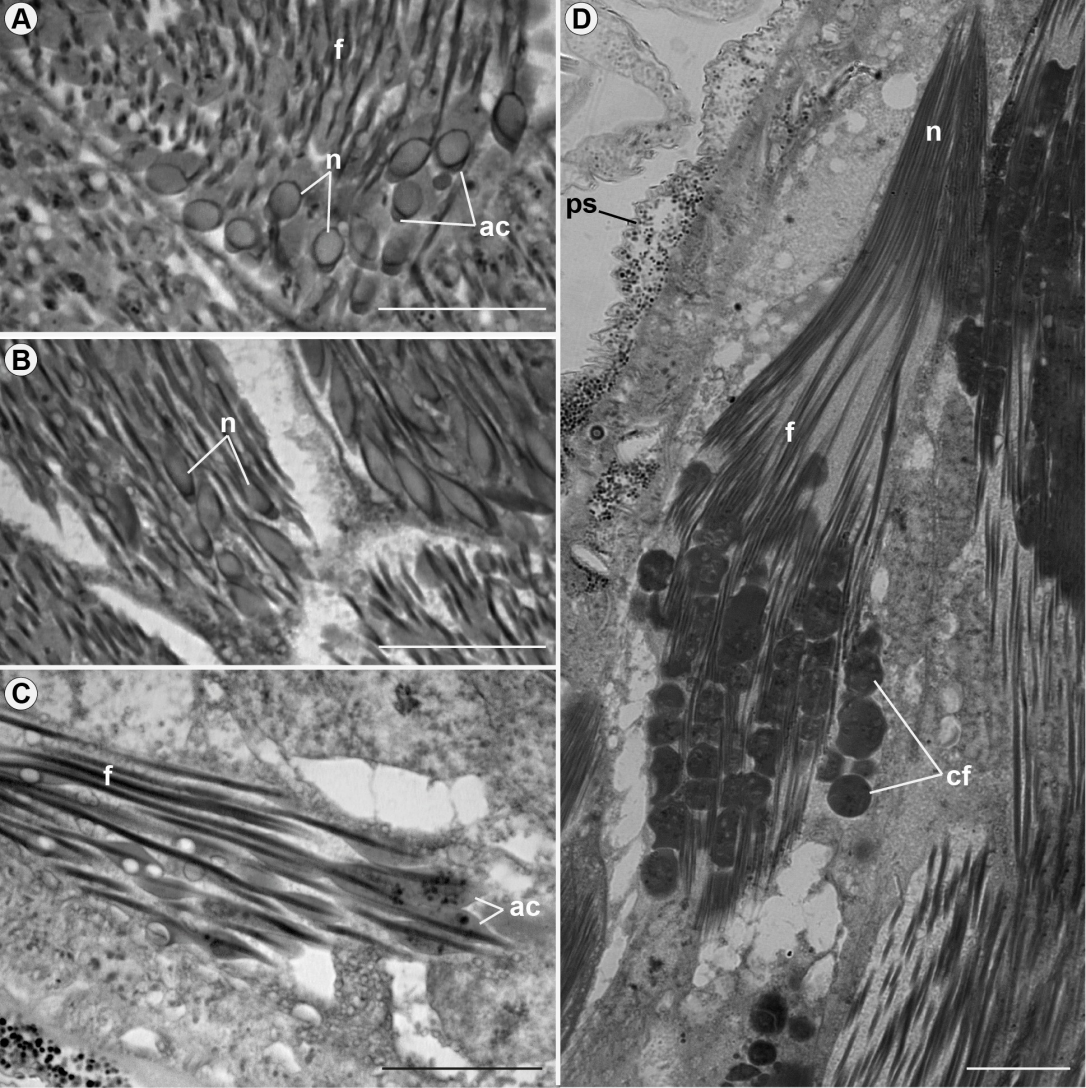
Details of spermiogenesis of spermatozoa type I in *Edessa rufomarginata*. Cysts with developing spermatids. In (A), (B), and (C), it is possible to observe the nuclei (n) lengthening and the acrosome (ac) positioning itself above the nucleus (n). In (D), the cyst is in the differentiation zone, where the nuclei (n) and flagella (f) elongate, and there is the presence of cellular fragments (cf) being eliminated. ps: peritoneal sheath. Scale bars: 20 μm.

**Fig 6:**
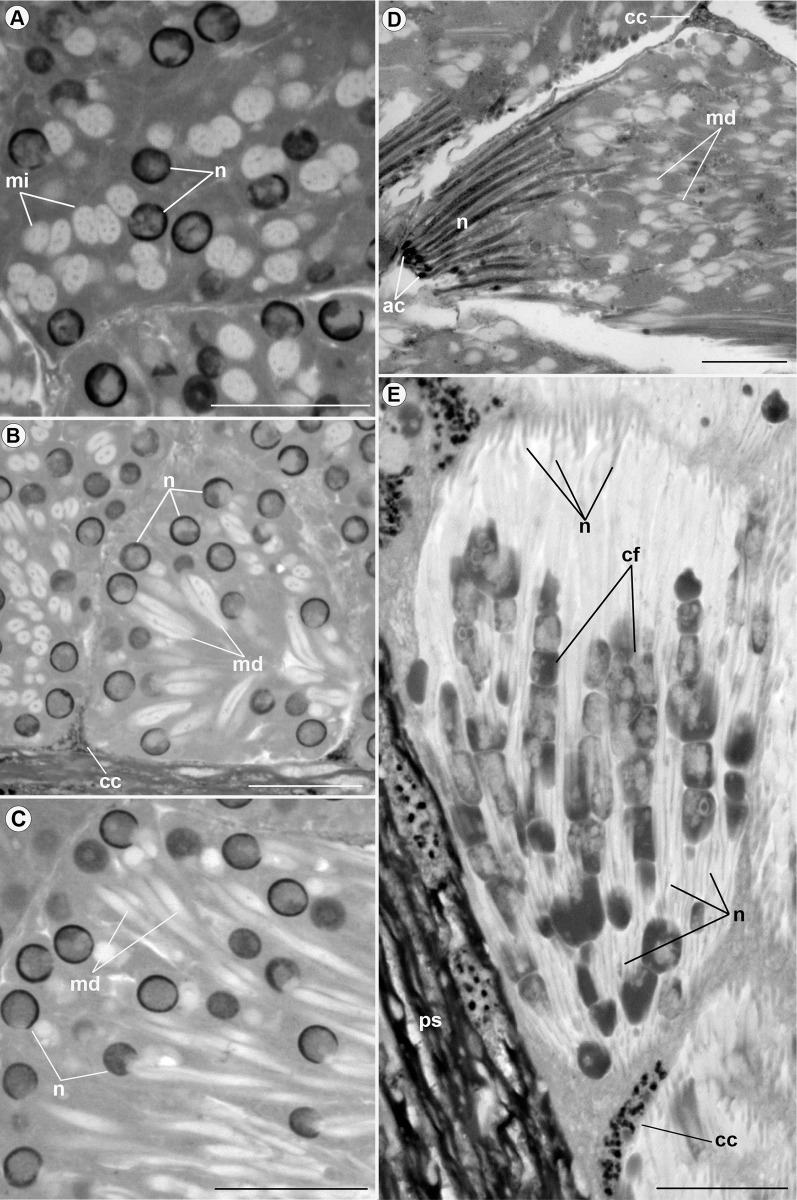
Details of spermiogenesis of spermatozoa type II *Edessa rufomarginata*. (A), (B), (C), and (D): cysts in the maturation zone, where mitochondria (mi) fuse. Note the rounded nuclei (n) in A-C. In B and C, nebenkerns fission occurs, giving rise to two mitochondrial derivatives (md). In (D), the nuclei (n) are elongated, and the acrosome (ac) and mitochondrial derivatives (md) are visible. In E, during the development process spermatozoa are in the differentiation zone. It is possible to observe sperm nuclei spread throughout the cyst and many cell fragments (cf) being eliminated. cc: cystic cell nucleus; ps: peritoneal sheath. Scale bars: 20 μm.

Both types of spermatozoa (I and II) similarly change their structural organization during the development. Thus, throughout the process, these changes can be visible in three zones of the testicular follicles: growth zone, maturation zone, and differentiation zone (Fig 3). In the growth zone, mitochondria fuse to form nebenkerns and elongate around the axoneme, forming mitochondrial derivatives (Figs 4B–4D and 6A–6D). The nuclei of spermatogonia, at the beginning of spermatogenesis, have a rounded shape (Figs 4A–4D and 6A–6C) and, in the maturation zone, the nucleus is elongated (Figs 5A–5C and 6D). In the differentiation zone, the nuclei and flagella complete their elongation. Spermatozoa have different ways of organizing themselves within cysts during spermiogenesis. Spermatozoa type I are organized neatly in the register in the cysts (Fig 5D), whereas spermatozoa type II appear disorganized within the cyst. In the latter type, sperm are positioned at varying heights and directions (Fig 6E). Many cell fragments were observed at the end of spermiogenesis (Figs 5D and 6E).

**Fig 7:**
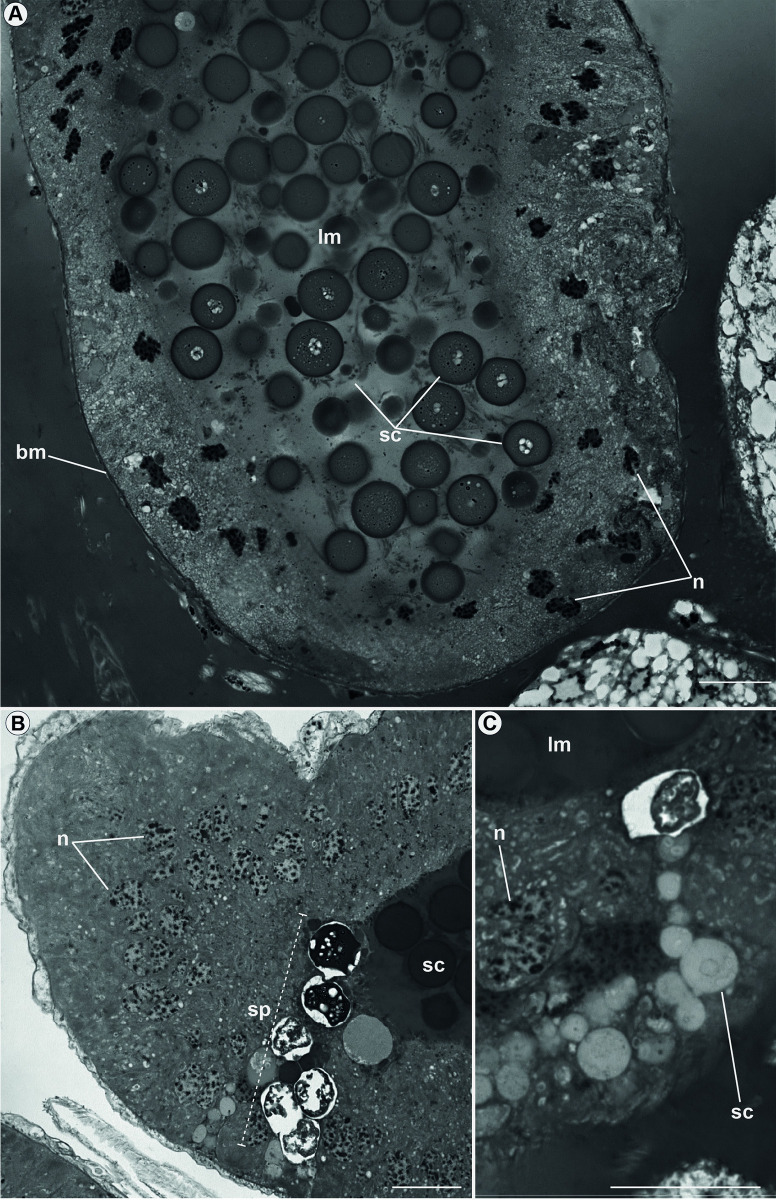
Accessory gland 1 (ag1) of *Edessa rufomarginata*. Details of the accessory gland of mesodermal origin. In (A), it is possible to observe many glycoprotein granules (sc) in the lumen (lm) of the gland. Epithelial cells do not present a specific organization, but the nuclei (n) are visible, with partially condensed chromatin and the basement membrane (bm). In (B), secretion (sp) production is visible in several epithelium cells and directed to the lumen (lm). The nuclei (n) present varied morphology and decondensed chromatin. In (C), several secretory vesicles (sc) are directed to the lumen (lm) of the gland. The nuclei (n) also vary morphologically, and their chromatin is decondensed. Scale bars: 20 μm.

Both accessory glands have high secretory activity, and different types of secretion can be observed. The pair of small glands (ag1) (Fig 1B) have mesodermal origin. These glands are formed by cubic epithelial cells with predominantly rounded nuclei and partially decondensed chromatin (Fig 7A–7C). Several secretion vesicles form in the cytoplasm and are directed towards the gland’s lumen. Using P.A.S. techniques, we identified that in these secretions predominate glycoproteins.

**Fig 8:**
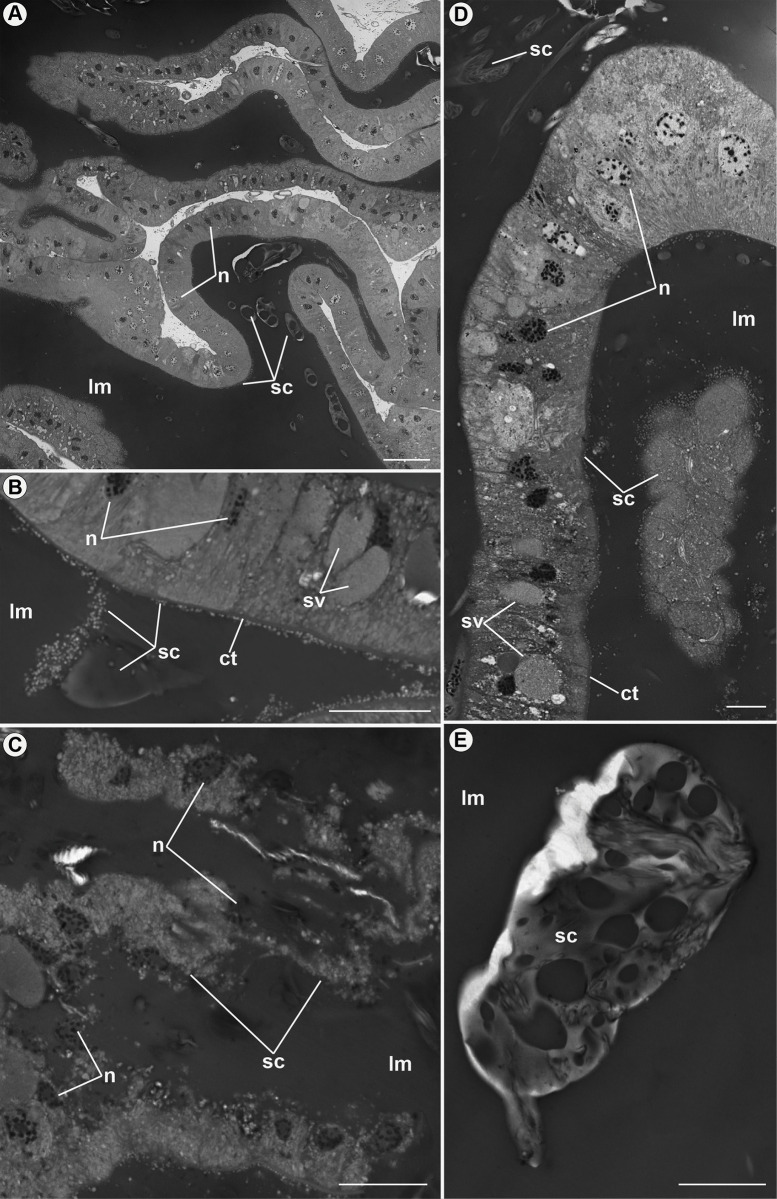
Accessory gland 2 (ag2) of *Edessa rufomarginata*. Details of the accessory gland of ectodermal origin. In (A) it is possible to observe the dense secretion (sc) in the lumen (lm) of the gland. Epithelial cells are elongated, with nuclei (n) close to the medial-basal region. Furthermore, nuclei (n) show variation in chromatin compaction. In (B) and (D), the secretion vesicles (sv) are visible in the cytosol of the cells, and their release (secretion: sc) into the lumen (lm) of the gland. After release, these compactors merge, forming larger mixtures. In both photos (B and D), the cuticle (ct) is evident, indicating the ectodermal origin of the gland. In (C), holocrine secretions (sc) are directed to the lumen (lm) of the gland. The nuclei (n) of the epithelial cells mix with the secretion (sc) in the lumen (lm) of the gland, demonstrating cellular disorganization at the time of secreting the products. (E): Detail of a secretory granule (sc) fused into the lumen (lm) of the gland. Scale bars: 20 μm.

The other type (ag2) is a single, and large accessory gland (Fig 1B), with high secretory activity (Fig 8A). A thin cuticle lining the glandular lumen (Fig 8B), indicating it has an ectodermal origin. The glandular tissue is formed by slightly elongated, cubic epithelial cells with rounded nuclei and chromatin varying in condensation (Fig 8A–8D). Furthermore, many secretory vesicles are observed in the cytoplasm of these cells (Fig 8C), and the lumen is filled with secretions (Fig 8A–8D). After being released, the secretions fuse, forming large dense particles (Fig 8A and 8C–8E). Meanwhile, in specific cells, it is possible to observe the entire cellular content being released in holocrine secretions, including the nucleus region (quite evident), into the lumen of the accessory gland (Fig 8B).

## Discussion

In this study, we report the presence of dimorphic spermatozoa in Edessinae for the first time, which means they have two morphotypes. The spermatozoa type I of *E*. *rufomarginata* is considered typical to fertilization, while type II is classified as aberrant. These aberrant sperm have unique morphological characteristics [22–24] and probably perform different reproduction functions or strategies during copulation distinct from fertilizing spermatozoa.

Production of more than one morphological type of spermatozoa has been reported in different species of Pentatomidae, specifically in Edessinae, Discocephalinae, and Pentatominae [11, 20–24, 26–29]. In Pentatomidae, the number of sperm primarily ranges from two (dimorphism) or three (polymorphism) types. Dimorphism predominantly occurs in this family in species with at least four testicular follicles, where the first three are typically responsible for producing fertilizing sperm, while the 4th generates aberrant sperm. Souza and Itoyama [28] numbered each follicle of *Euschistus heros* (Fabricius, 1798) according to its position in relation to the vas deferens. In this case of this study, follicle 1 of *E*. *ruformaginata* is the one closest to the exit of the vas deferens, and the 4th is the one farthest away. Interestingly, *Edessa bifida* (Say, 1832) accounted for 5 testicular follicles, with follicles 2 and 4 serving as the site for producing typical larger cells, according to cytogenetic data [20]. This finding catches our attention since both *E*. *bifida* and *E*. *rufomarginata* belong to the same genus, and so far, none of the studies have reported the occurrence of abnormal meiosis in follicle 2, only from follicle 4 onwards. Recently, Santos et al. [30] suggested the relocation of *E*. *bifida* to the genus *Ascra*, based on morphological characteristics mainly of the genitalia. However, data on the male reproductive system and spermatozoa of *E*. *bifida* (*Ascra bifida*) have not yet been published. Due to this discrepancy in information regarding such closely related species, we consider verifying the data in question essential. The difference in the number of follicles and the presence or absence of aberrant sperm in this species may support the genus change.

Sperm polymorphism has generally been documented in heteropterans possessing 5 to 8 testicular follicles. Typically, two follicles produce longer sperm (4 and 6), while a single follicle produces shorter sperm (5), with the remaining follicles producing intermediate-size gametes (1, 2, and 3, or even 7 and 8). Notably, sperm size does not correlate with lobe size; sometimes, the follicle that produces shorter sperm is the largest, while those that produce longer sperm are the smallest ([22–24, 26, 27]. The "harlequin" lobe, named for variation during normal meiosis, corresponds to the largest follicle in genus *Loxa* Amyot & Serville, 1843, the testicular follicle 5 [24, 31], which is responsible for the production of short sperm. In *Arvelius albopunctatus* (De Geer, 1773), follicles 3 and 5 are smaller and responsible for producing longer sperm, while follicle 4 is the largest and produces short sperm [27]. Similar changes occur during the development of *E*. *rufomarginata* sperm cells; however, even though meiosis produces aberrant sperm, such as Type II, they are produced in the smallest lobe, the testicular follicle 4, different from what was previously described.

The aberrant Type II sperm produced by *E*. *rufomarginata* may play different roles during reproduction. We observed that aberrant sperm had greater motility compared to typical sperm, this could indicate that these type II spermatozoa possibly help in the transfer of type I spermatozoa [32]. Interestingly, in the spermatheca of *E*. *rufomarginata* females, we exclusively observed the longer and typical sperm (type I). In this sense, short sperm are generally not used to fertilize eggs. However, according to Schrader [22] and Swallow & Wilkinson [24], shorter sperm (with a smaller number of chromosomes than the usual complement) do not leave the male reproductive system or, at least, were not found in females. Based on this information, perhaps aberrant Type II sperm can be rapidly degraded in the female reproductive system. Schrader [22, 23, 31] raises hypotheses about the persistence of the production of sperm not used in fertilization. He proposed that they could provide supplemental nutrients, particularly nucleoproteins, to the developing egg, fertilizing sperm or to be used for sperm before being transferred to the female [24]. Other hypotheses include the role of aberrant spermatozoa in sperm competition [32–35]. If aberrant sperm reached the female reproductive system, non-fertilizing sperm could be used to fill the spermatheca or copulatory bursa of females, preventing other males from depositing their sperm (see [35, 36]). It could also increase the chance of paternity of this first male or even provide nutrients to females [32, 35].

Regardless of the function that aberrant sperm may play in the male and female reproductive physiology, its morphological characteristics are undoubtedly important for phylogeny and taxonomy. It is notable that, although dimorphic spermatozoa differ mainly in total size or nucleus size, they most often present a very similar morphology when observed under light microscopy [11, 24, 37]. However, the aberrant type II sperm of *E*. *rufomarginata* possess a distinctive and easily identifiable morphology compared to type I. Type II sperm is slightly flattened and is significantly shorter than typical fertilizing sperm. Notably, the nuclei of aberrant sperm are longer than those of typical sperm, covering more than half of their total length. Its nuclear characteristics, such as changes in chromatin compaction and its insertion into elements of the flagellum, further highlight its differentiated morphology.

The anatomy of the male reproductive system of *E*. *rufomarginata* is similar to other Pentatomidae, following their general pattern of organization. The testes are elongated as in *Chinavia ubica* Rolston, 1983, *Oebalus insularis* Stål, 1872, *Piezodorus guildinii* (Westwood, 1837) [15], and *Apodiphus amygdali* (Germar, 1817) [13]. However, some pentatomids may have elongated ovoid testes, as in *Nezara viridula* Linnaeus, 1758 [38], or even kidney-shaped in *Halys dentatus* (Fabricius, 1775) [39]. Other variations are in the color of the peritoneal sheath, being yellowish, red, or even orange [8, 13–15, 28, 40]. *E*. *rufomarginata* has four testicular follicles, few when compared to other Pentatomidae, which usually have seven follicles [16, 22–24]. These data can be helpful in taxonomy since the characteristics mentioned above tend to be conserved within a species, genus, or even family [8, 16, 41].

*E*. *rufomarginata* has two accessory glands that produce different types of secretions. In accessory gland 1, the secretions of merocrine type are predominantly glycoproteins, while in the accessory gland 2 were not positives to glycoprotein test and the secretions seem to be merocrine and holocrine. According to Happ [42], holocrine secretions are less common in insects, and they are reported in *Drosophila melanogaster* (Meigen, 1830) [43] and *Musca domestica* Linnaeus, 1758 [44], for example. The merocrine and apocrine secretions are the most frequently observed in insects [42]. This secretion can help in the formation of the mating plug in females through the polymerization of smaller particles [42, 45], thus preventing sperm from subsequent copulations [46] from reaching the female’s copulatory bursa and/or spermatheca. Therefore, accessory glands are sources of many glycoproteins and lipids that can trigger changes in the physiology and behavior of males and females [35, 47–50]. Overall, it increases the chances of fertilization by the male, giving it advantages in sperm competition.

Based on these distinctive characteristics, it is reasonable to infer that if the morphology of spermatozoa commonly used in fertilization is already helpful for taxonomic purposes, a species that presents two types of spermatozoa further enhances the morphological characteristics in the analyses. Additionally, we highlight that the type II sperm of *E*. *rufomarginata*, which has such a peculiar and aberrant morphology, will have a higher value for taxonomic purposes than those dimorphic spermatozoa considered typical that vary, most often, in total and nuclear length. Notably, the observations raised here will significantly contribute to establishing taxonomic limits in a group as chaotic as *Edessa*, or at least for the subfamily Edessinae.

## Final considerations

In summary, our investigation into the reproductive system of *E*. *rufomarginata* reveals the presence of dimorphic spermatozoa, one typical and the other with an aberrant morphotype. The aberrant spermatozoa may play diverse functions in reproduction other than fertilization. Furthermore, the male reproductive system presents unique anatomical characteristics with taxonomic importance. We also highlight that the fourth testicular follicle presents a distinct spermiogenesis and pattern of organization in the cysts, differing from the other follicles and being responsible for producing the aberrant sperm morphotype.

The observed sperm dimorphism aligns with existing theories and contributes to our knowledge of Pentatomidae’s reproductive strategies. Future works, including ultrastructural analysis, may further complement the data collection of this very diverse group. These findings highlight the importance of understanding the characteristics of the male reproductive system in insect biology.
